# Outcomes in an educational skin‐model session on point‐of‐care ultrasound for diagnosing calciphylaxis

**DOI:** 10.1002/ski2.467

**Published:** 2024-10-28

**Authors:** Max E. Oscherwitz, Christopher T. Kelly, Bridget Francis, Casey Glass, John R. Edminister, Steven Feldman, Lindsay C. Strowd

**Affiliations:** ^1^ Center for Dermatology Research Department of Dermatology Wake Forest School of Medicine Winston‐Salem North Carolina USA; ^2^ UAB Heersink School of Medicine Birmingham Alabama USA; ^3^ Department of Internal Medicine Wake Forest School of Medicine Winston‐Salem North Carolina USA; ^4^ Wake Forest School of Medicine Center for Experiential and Applied Learning Winston‐Salem North Carolina USA; ^5^ Department of Emergency Medicine Wake Forest School of Medicine Winston‐Salem North Carolina USA; ^6^ Department of Pathology Wake Forest School of Medicine Winston‐Salem North Carolina USA; ^7^ Department of Social Sciences & Health Policy Wake Forest School of Medicine Winston‐Salem North Carolina USA; ^8^ Department of Dermatology University of Southern Denmark Odense Denmark

Dear Editor,

Calciphylaxis is a disorder of vascular calcification that is generally seen in patients with kidney failure.^1^, ^2^ Two sub‐types of calciphylaxis exist: uraemic and non‐uraemic. Uraemic calciphylaxis exclusively affects patients with advanced end‐stage renal disease (ESRD) and has an incidence of 35 cases per 10,000 patients receiving dialysis in the United States.^2^, ^3^ Although the incidence of non‐uraemic calciphylaxis is not known, it appears rare and may be found in patients with normal renal function, less advanced ESRD, and other conditions including hyperparathyroidism, malignant neoplasm, alcoholic liver disease and autoimmune disorders.^2^, ^3^

If not diagnosed and treated promptly, the disease can result in significant tissue damage and has a 1‐year mortality rate of over 50%.^4^ Current techniques used to diagnose calciphylaxis rely heavily on clinical suspicion and skin biopsy; however, limitations associated with histopathologic examination limit the utility of this diagnostic tool.^2^

Point‐of‐care ultrasound (POCUS) has emerged as a powerful and non‐invasive diagnostic tool utilized by multiple medical subspecialities. POCUS has many advantages, including ease of use, cost‐effectiveness and lack of ionizing radiation. Advances in technology have refined POCUS's ability to visualize the skin and cutaneous pathology. POCUS is now utilized to detect cysts, tumours, vascular lesions and inflammatory conditions, and recent studies have described the diagnostic potential of POCUS for calciphylaxis.^2^, ^4^, ^5^, ^6^ Here, we describe the outcomes of an educational session on POCUS for diagnosing calciphylaxis among dermatology residents.

## 1 METHODS

Twelve dermatology residents attended a two‐part workshop facilitated by dermatology, internal medicine and emergency medicine faculty. They first participated in a lecture on calciphylaxis pathophysiology and clinical presentation, as well as POCUS techniques and principles. Immediately following this didactic content, they participated in a hands‐on station‐based workshop where they were able to practice scanning using different high‐frequency ultrasound machines on normal skin, calciphylaxis skin models and water bath scanning of digits. The calciphylaxis skin model was previously created by the authors and mimics arterioles lined with calcium using ballistic gel, fishing line and crushed‐up seashells (Supplemental Figure 1a,b). Residents were given a pre‐ and post‐workshop survey to assess their attitudes towards POCUS as a diagnostic modality and efficacy of the workshop. Statistical analysis was conducted via Fisher’s exact test.

## 2 RESULTS

Nine and 12 residents completed the pre‐ and post‐workshop survey, respectively. The components of resident performance were analyzed by subjective (Table 1) and objective (Table 2) measures. Regarding subjective performance, residents' attitudes shifted towards higher rates of agreement for utilizing POCUS in a dermatologic setting, feeling more comfortable operating and interpreting results from POCUS studies and being more likely to consider utilizing POCUS in cases of suspected calciphylaxis after participating in the workshop. Regarding objective performance, residents demonstrated improvement in all assessment categories apart from their ability to describe a characteristic POCUS finding in calciphylaxis.

**TABLE 1:** Attitudes towards POCUS and calciphylaxis.

Statement	Pre‐workshop	Post‐workshop	Fisher exact test
POCUS is a valuable diagnostic tool in the outpatient dermatology setting	SA: 0/9 (0%)	SA: 2/12 (16.7%)	*p* = 0.0006*
	A: 2/9 (22.2%)	A: 10/12 (83.3%)	
	N: 4/9 (44.4%)	N: 0/12 (0%)	
	D: 3/9 (33.3%)	D: 0/12 (0%)	
	SD: 0/9 (0%)	SD: 0/12 (0%)	
POCUS is a valuable diagnostic tool in the inpatient dermatology setting	SA: 0/9 (0%)	SA: 10/12 (83.3%)	*p* = 0.0004*
	A: 8/9 (88.9%)	A: 2/12 (16.7%)	
	N: 1/9 (11.1%)	N: 0/12 (0%)	
	D: 0/9 (0%)	D: 0/12 (0%)	
	SD: 0/9 (0%)	SD: 0/12 (0%)	
I feel confident in my ability to correctly perform a POCUS scan of skin/soft tissue	SA: 0/9 (0%)	SA: 1/12 (8.3%)	*p* = 0.0002*
	A: 1/9 (11.1%)	A: 8/12 (66.7%)	
	N: 0/9 (0%)	N: 3/12 (25%)	
	D: 6/9 (66.7%)	D: 0/12 (0%)	
	SD: 2/9 (22.2%)	SD: 0/12 (0%)	
I feel confident in my ability to correctly interpret skin and soft tissue ultrasound findings	SA: 0/9 (0%)	SA: 1/12 (8.3%)	*p* < 0.0001*
	A: 0/9 (0%)	A: 7/12 (58.3%)	
	N: 1/9 (11.1%)	N: 4/12 (33.3%)	
	D: 6/9 (66.7%)	D: 0/12 (0%)	
	SD: 2/9 (22.2%)	SD: 0/12 (0%)	
I feel confident in my ability to operate an ultrasound machine by controlling depth and gain for image optimization	SA: 0/9 (0%)	SA: 5/12 (41.2%)	*p* = 0.0003*
	A: 1/9 (11.1%)	A: 7/12 (58.3%)	
	N: 2/9 (22.2%)	N: 0/12 (0%)	
	D: 3/9 (33.3%)	D: 0/12 (0%)	
	SD: 3/9 (33.3%)	SD: 0/12 (0%)	
I feel confident in my ability to use POCUS to augment my history and traditional physical exam to obtain additional clinically relevant information that can be incorporated into my clinical reasoning	SA: 0/9 (0%)	SA: 3/12 (25%)	*p* = 0.0011*
	A: 1/9 (11.1%)	A: 7/12 (58.3%)	
	N: 1/9 (11.1%)	N: 2/12 (16.7%)	
	D: 6/9 (66.7%)	D: 0/12 (0%)	
	SD: 1/9 (11.1%)	SD: 0/12 (0%)	
I would consider utilizing POCUS in any case of suspected dermatologic disease to help in my clinical evaluation and assessment	SA: 1/7 (14.3%)	SA: 2/12 (16.7%)	*p* = 0.075
	A: 1/7 (14.3%)	A: 8/12 (66.7%)	
	N: 3/7 (42.9%)	N: 1/12 (8.3%)	
	D: 2/7 (28.6%)	D: 1/12 (8.3%)	
	SD: 0/7 (0%)	SD: 0/12 (0%)	
	^2 responses left blank		
I would consider utilizing POCUS in cases of suspected calciphylaxis to assist in the clinical evaluation, diagnostic process and management decisions	SA: 5/9 (55.6%)	SA: 10/12 (83.3%)	*p* = 0.33
	A: 4/9 (44.4%)	A: 2/12 (16.7%)	
	N: 0/9 (0%)	N: 0/12 (0%)	
	D: 0/9 (0%)	D: 0/12 (0%)	
	SD: 0/9 (0%)	SD: 0/12 (0%)	
I am interested in receiving further specialized training in the use of POCUS to help identify other dermatological diseases	SA: 2/9 (22.2%)	SA: 5/12 (41.2%)	*p* = 0.44
	A: 4/9 (44.4%)	A: 6/12 (50%)	
	N: 3/9 (33.3%)	N: 1/12 (8.3%)	
	D: 0/9 (0%)	D: 0/12 (0%)	
	SD: 0/9 (0%)	SD: 0/12 (0%)	

Abbreviations: A, Agree; D, Disagree; N, Neutral; SA, Strongly agree; SD, Strongly Disagree.

*Statistically significant.

**TABLE 2:** Knowledge of POCUS and calciphylaxis concepts.

Question topic	Pre‐workshop # correct	Post‐workshop # correct	Fisher exact test
Ability to define general echogenicity	6/8 (75%) (1 left blank)	9/12 (75%)	*p* = 1.0
Ability to define hyperechogenicity and hypoechogenicity	7/9 (77.8%)	11/12 (91.2%)	*p* = 0.55
Ability to choose which POCUS probe would be most appropriate for examining a patient in the case of suspected calciphylaxis	4/9 (44.4%)	12/12 (100%)	*p* = 0.005*
Ability to discern the optimal POCUS depth to look for affected vessels in calciphylaxis	7/9 (77.8%)	12/12 (100%)	*p* = 0.17
Ability to discern a characteristic POCUS finding in calciphyalxis	8/9 (88.9%)	9/12 (75%)	*p* = 0.60
Ability to ID the presence of calciphylaxis when shown a POCUS image (with at least 50% accuracy)	3/9 (33.3%)	8/12 (66.7%)	*p* = 0.19
Ability to ID the absence of calciphylaxis when shown a POCUS image (with at least 50% accuracy)	3/9 (33.3%)	5/12 (41.2%)	*p* = 1.0

*Statistically significant.

## 3 DISCUSSION

Calciphylaxis is a disease which requires expedient and accurate diagnosis to minimize morbidity and mortality. POCUS, while historically underutilized in dermatology, is gaining popularity for cutaneous use as the technology advances and resolution of skin structures improves. Due to its non‐invasive nature, POCUS may be an optimal tool to detect calciphylaxis, especially given the classic signs of hyperechoic calcium in dermal vessels coupled with a hypoechoic shadow. (Supplemental Figure 2a,b) Barriers to use POCUS include accessibility of high‐frequency ultrasound machines and lack of training in dermatology programmes.

The residents who attended the workshop had a range of POCUS experience (no formal training to medical school longitudinal training). The main objective of the workshop was to introduce basic concepts and techniques of POCUS and train residents on how to identify ultrasonographic features of calciphylaxis. The workshop was a success as residents reported enjoying the team‐building and hands‐on components of the workshop, and their survey results reflect an improvement in their attitudes, confidence and knowledge regarding ultrasound, calciphylaxis and the ability to detect calciphylaxis using POCUS.

The study's principal limitation was its small sample size, which resulted in low statistical power. Furthermore, the model we created has not been formally validated as a diagnostic tool for calciphylaxis, emphasizing the importance of using POCUS as a supplemental tool. We recommend the utilization of POCUS in combination with other diagnostic modalities (i.e., skin biopsy, the current gold standard of diagnosis) when assessing for calciphylaxis. Future studies may incorporate data from multiple sites (i.e., a summation of multiple residency programmes' results) and longitudinal workshop training.

## CONFLICT OF INTEREST STATEMENT

Steven Feldman has received research, speaking and/or consulting support from a variety of companies including Galderma, GSK/Stiefel, Almirall, Leo Pharma, Boehringer Ingelheim, Mylan, Celgene, Pfizer, Valeant, Abbvie, Samsung, Janssen, Lilly, Menlo, Merck, Novartis, Regeneron, Sanofi, Novan, Qurient, National Biological Corporation, Caremark, Advance Medical, Sun Pharma, Suncare Research, Informa, UpToDate and National Psoriasis Foundation. He is founder and majority owner of www.DrScore.com and founder and part owner of Causa Research, a company dedicated to enhancing patients' adherence to treatment. Lindsay Strowd has received research, speaking and/or consulting support from Galderma, Pfizer, Sanofi, Regeneron. The remaining authors have no conflicts to disclose.

## AUTHOR CONTRIBUTIONS

**Max E. Oscherwitz**: Conceptualization (equal); data curation (equal); formal analysis (equal); funding acquisition (equal); investigation (equal); methodology (equal); project administration (equal); resources (equal); validation (equal); visualization (equal); writing—original draft (equal); writing—review and editing (equal). **Christopher T. Kelly**: Conceptualization (lead); data curation (equal); formal analysis (equal); funding acquisition (lead); investigation (lead); methodology (lead); project administration (lead); resources (equal); software (equal); supervision (equal); validation (equal); visualization (equal); writing—original draft (equal); writing—review and editing (equal). **Bridget Francis**: Conceptualization (equal); funding acquisition (equal); investigation (equal); project administration (equal); writing—original draft (equal); writing—review and editing (equal). **Casey Glass**: Project administration (equal); writing—original draft (equal); writing—review and editing (equal). **John R. Edminister**: Project administration (equal); writing—original draft (equal); writing—review and editing (equal). **Steven Feldman**: Project administration (equal); writing—original draft (equal); writing—review and editing (equal). **Lindsay C. Strowd**: Conceptualization (lead); data curation (equal); formal analysis (equal); funding acquisition (lead); investigation (lead); methodology (lead); project administration (lead); resources (equal); software (equal); supervision (equal); validation (equal); visualization (equal); writing—original draft (equal); writing—review and editing (equal).

## FUNDING INFORMATION

This article received no specific grant from any funding agency in the public, commercial, or not‐for‐profit sectors.

## ETHICS STATEMENT

Not applicable.

## PATIENT CONSENT

Not applicable.

## Supporting information

Supporting Information S1

## Data Availability

Research data are not shared.
