# Supplementary material for: Outcomes in an educational skin‐model session on point‐of‐care ultrasound for diagnosing calciphylaxis
Source: Skin Health Dis. 2024 Oct 28;4(6):e467. doi: 10.1002/ski2.467 (PMC11608863; doi:10.1002/ski2.467)

**Supplemental Figure 1a (left), 1b (right):** The calciphylaxis skin model was created by the authors with the intent to mimic arterioles lined with calcium. Several prototypes were created using ballistic gel, fishing line, and crushed-up seashells (left) before the appropriate texture was attained. Dye was subsequently added to mimic skin and conceal the inserts (right).

**
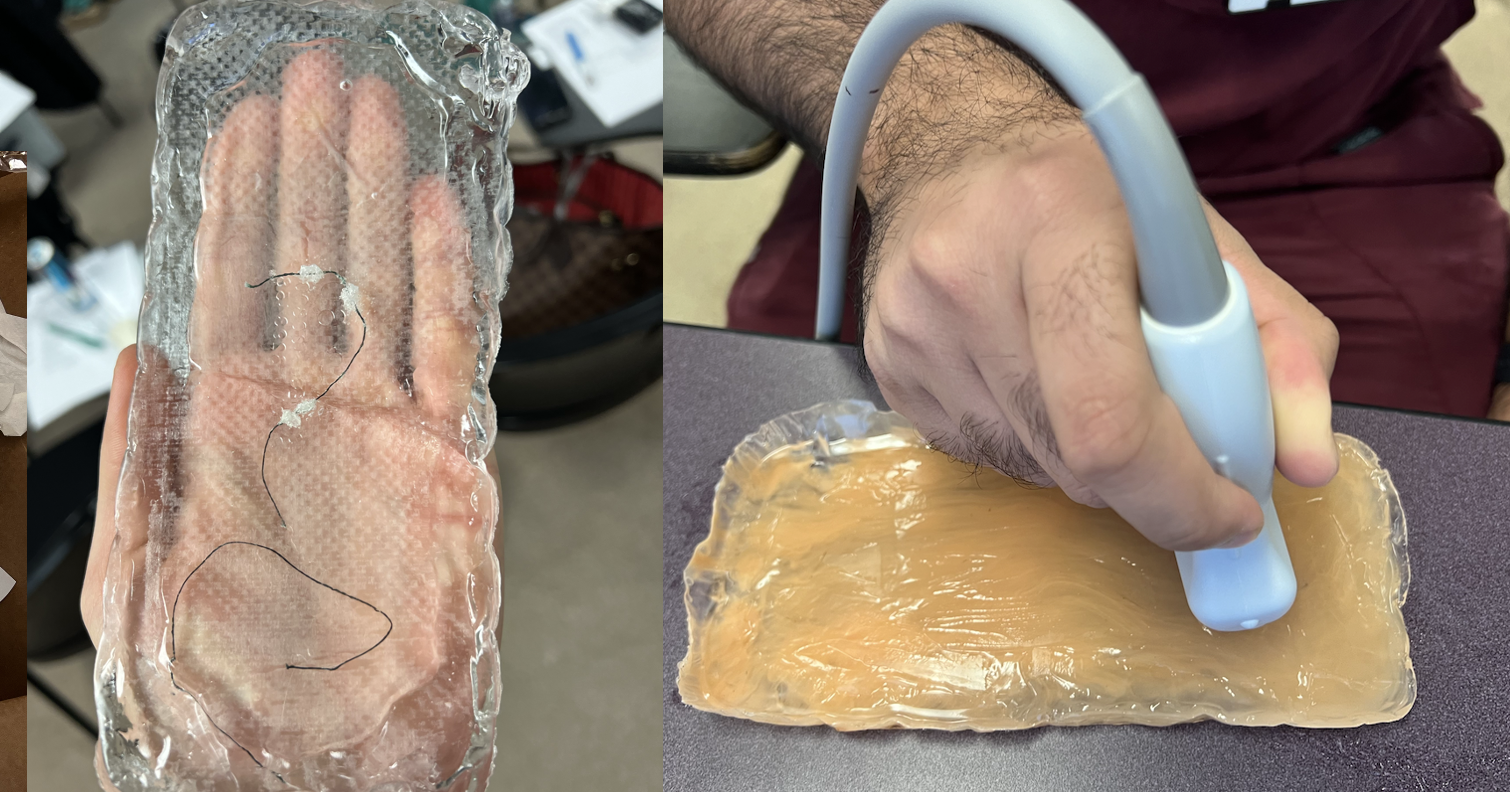
**

**Supplemental Figure 2a (left), 2b (right):** The characteristic finding of hyperechoic calcium in dermal vessels coupled with a hypoechoic shadow (red arrows) depicted in a side-by-side comparison of calciphylaxis in an actual patient (left) next to calciphylaxis depicted in the model (right).


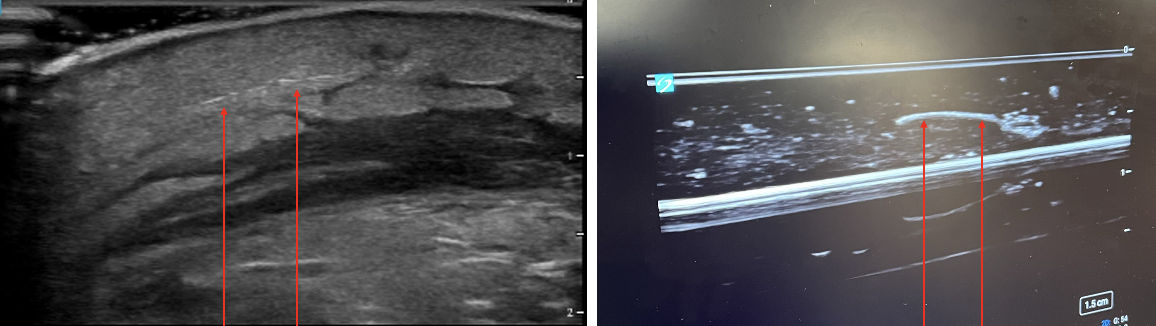

Supplement: Supplementary file 1 — Supporting Information S1 [file SKI2-4-e467-s001.docx]
